# Accuracy of Nanoscale Pitch Standards Fabricated by Laser-Focused Atomic Deposition

**DOI:** 10.6028/jres.108.0010

**Published:** 2003-04-01

**Authors:** Jabez J. McClelland, William R. Anderson, Curtis C. Bradley, Mirek Walkiewicz, Robert J. Celotta, Erich Jurdik, Richard D. Deslattes

**Affiliations:** National Institute of Standards and Technology, Gaithersburg, MD 20899-8412 USA; Research Institute for Materials, University of Nijmegen, NL-6535 ED Nijmegen, the Netherlands; National Institute of Standards and Technology, Gaithersburg, MD 20899-8412 USA

**Keywords:** atom optics, atomic ruler, chromium lines, laser focusing, nanoscale standards, nanotechnology, pitch standards

## Abstract

The pitch accuracy of a grating formed by laser-focused atomic deposition is evaluated from the point of view of fabricating nanoscale pitch standard artifacts. The average pitch obtained by the process, nominally half the laser wavelength, is simply traceable with small uncertainty to an atomic frequency and hence can be known with very high accuracy. An error budget is presented for a Cr on sapphire sample, showing that a combined standard uncertainty of 0.0049 nm, or a relative uncertainty of 2.3 × 10^−5^, is readily obtained, provided the substrate temperature does not change. Precision measurements of the diffraction of the 351.1 nm argon ion laser line from such an artifact are also presented. These yield an average pitch of (212.7777 ± 0.0069) nm, which agrees well with the expected value, as corrected for thermal contraction, of (212.7705 ± 0.0049) nm.

## 1. Introduction

With the drive toward miniaturization of a wide range of technologies, in fields ranging from electronics, to magnetics, to chemistry and biotechnology, dimensional metrology on the nanometer scale has become an increasingly important area for research. One of the key elements of dimensional measurement on any scale is the development of length standards of appropriate dimensions. If well-characterized devices are to be manufactured reproducibly, it is important to be able to measure them with confidence, and this involves using well-understood length standards on a suitable scale. On the nanometer scale, length standards pose particular challenges because many effects such as thermal expansion, material graininess, and material creep and relaxation become dominant. In addition, it is difficult to transfer a well-defined macroscopic length standard to the nanometer scale because uncertainties that may be insignificant on the larger scale can become dominant in the transfer process.

In this paper we examine the accuracy of artifacts made by laser-focused atomic deposition as a first step in establishing their suitability for use as nanoscale pitch standards. Artifacts made by this method are particularly interesting from a standards point of view because they can take the form of a highly regular array of lines (or dots) whose average pitch, or spacing between the features, can be traced directly to an atomic transition frequency. Atomic frequencies are useful as absolute standards because they can be measured with extremely high accuracy and they are minimally perturbed by environmental conditions. For this reason, for example, they are the reference of choice in developing time standards via atomic clocks [1]. The connection between laser-focused atomic deposition and atomic frequencies thus opens the possibility of creating a nanoscale length standard that is traceable to a highly accurate, constant, physically measurable quantity.

**Fig. 1:**
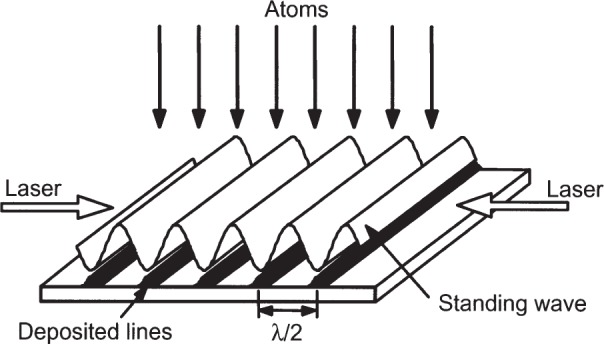
Schematic of laser-focused atomic deposition process, showing incident atoms, laser standing wave, and deposited lines.

Laser-focused atomic deposition is a fabrication technique in which atoms are evaporated onto a substrate through a standing wave of laser light that propagates parallel to, and just above, the substrate surface (see Fig. 1). The laser light is nominally single-frequency (linewidth 1 MHz or less) and stabilized at a frequency near a strong optical absorption line in the atom. With suitable choice of laser intensity (usually a few megawatts per square meter) and detuning from the atomic line center (usually a few hundred megahertz), the nodes of the standing wave will act as an array of “lenses” for the atoms, concentrating them into an array of lines on the substrate. The result is essentially a “contact print” of the laser standing wave in the form of a grating with a pitch of a few hundred nanometers and lines a few tens of nanometers wide.

Because the laser-focused atomic deposition process relies on a resonant interaction between the laser and the atom, the pattern will not form unless the laser frequency is within a few hundred megahertz of the atomic resonance. Thus the laser frequency is always within a fraction of 1 × 10^−6^ of the atomic resonance frequency (typically several times 10^14^ Hz). In fact, modern spectroscopic methods make it relatively easy to measure the frequency of a laser relative to an atomic resonance with quite high accuracy, so that any uncertainty in the laser frequency is almost completely dominated by uncertainty in the knowledge of the exact atomic resonance frequency. The wavelength *λ* of the laser light is related to the laser frequency *ν* by *λ = c/*(*nν*) where *c* is the vacuum speed of light, defined to be exactly 299 792 458 m · s^−1^, and *n* is the local index of refraction. Because the depositions are carried out in a very high vacuum, *n* is extremely close to unity. It follows that the standing wave, which has a period *λ*/2 (with some very small corrections; see below), has a periodicity that is essentially as well determined as the atomic frequency. Since the standing wave is nominally parallel to the surface of the substrate during deposition, this degree of certainty transfers nearly perfectly to the deposited structure, resulting in an inherently well-characterized pitch artifact. Of course, there are details of the deposition process involving alignment and other physical effects that have an influence on the ultimate pitch of the resulting artifact. These will be the subject of Sec. 2 below. As will be seen, however, these effects are generally quite small and it is thus relatively straightforward to fabricate an artifact with relative uncertainty in average pitch of a few times 10^−5^.

Demonstrations of laser-focused atomic deposition have been carried out with a number of atomic species, including sodium[2], chromium[3,4] and aluminum[5]. A related process, in which a laser standing wave focuses neutral atoms onto a resist and the pattern is transferred by etching has also been demonstrated with cesium atoms[6] and metastable neon atoms[7]. Since the first demonstrations, a number of extensions of the process have been investigated, including two-dimensional patterning[8,9], patterning at one fourth the pitch[10], etching the pattern into the substrate[11], and beating of two nearly equal patterns to form a long-period artifact[12]. Some metrological aspects of the process have also been discussed[13]. A general review of nanofabrication with atom optical techniques can be found in reference [14].

## 2. Pitch Corrections and Uncertainties

In this section we analyze the possible systematic errors and uncertainties that affect the average pitch of an artifact created by laser-focused atomic deposition. For concreteness, we discuss these in the detailed context of depositions carried out with Cr (*λ* = 425.5533 nm) on a sapphire substrate in the NIST apparatus in February of 1998. However, most of the effects discussed are generic and can be transferred to other deposition scenarios with appropriate scaling of the relevant parameters. An attempt has been made to be thorough in listing all the possible sources of corrections and uncertainties. It must be acknowledged, though, that it is possible that one or more effects may have been overlooked. These effects, if they exist, will have to await discovery in further research. A summary of the corrections and uncertainties discussed below is shown in Table 1. The result of this study is that the pitch of the artifact under consideration is 212.7787 nm with a combined standard uncertainty of 0.0049 nm, provided it is maintained at a temperature of 29 °C (see Sec. 2.5).

Because the uncertainties described in this section are based on such sources as auxiliary measurements, manufacturers’ specifications, and reasonable estimates, they are considered as type B[15]. They are intended to be interpreted as one standard deviation, although a precise quantification in terms of standard deviation is not strictly speaking possible in some cases.

### 2.1 Wavelength Effects

The most obvious factor controlling the pitch of an artifact produced by laser-focused atomic deposition is the wavelength *λ* of the laser light used. As discussed above, the wavelength is directly related to the frequency of the laser light *ν* and the local index of refraction *n* by *λ = c/*(*nν*). Any correction or uncertainty in *λ* is thus governed by corrections and uncertainties in *ν* and *n*.

#### 2.1.1 Laser Frequency Uncertainty

The laser frequency *ν* is generally determined without any significant corrections, but, of course, with some degree of uncertainty. This uncertainty has three components, which arise from the way in which *ν* is set. Typically the laser output is split into two beams by an acousto-optic modulator. These two beams are frequency shifted by several hundred megahertz relative to each other as a result of the radio frequency (RF) signal driving the modulator. One of them is used to form the standing wave, while the other is used to collimate the atom beam via laser cooling. This cooling beam is locked one to two atomic linewidths below the atomic resonance. The uncertainty of the laser frequency is therefore governed by (1) the uncertainty in the RF frequency, (2) the accuracy of the frequency lock relative to the atomic transition, and (3) the uncertainty in the absolute atomic frequency. The first two of these uncertainties are a matter of the technical performance of the apparatus, while the last relies on an independent spectroscopic measurement of the energy difference between the two atomic energy levels involved in the interaction with the laser.

For the NIST depositions, the RF generator frequency was specified to be accurate to ± 75 kHz, resulting in a laser frequency relative uncertainty contribution of ± 1.1×10^−10^ and a corresponding pitch uncertainty of ± 2.3 10^−8^ nm. The atomic resonance lock was accurate to ± 2 MHz, contributing a relative uncertainty of ± 2.8×10^−9^, or a pitch uncertainty of ± 6.0 × 10^−7^ nm. The energy levels involved in the atomic transition were the ^7^S_3_ and ^7^P_4_° states in Cr, the energy spacing of which has been reported in wavenumbers as 23 498.821 ± 0.005 cm^−1^[16]. From this we find that the absolute atomic resonance frequency uncertainty is ± 150 MHz, which translates into a ± 4.5 × 10^−5^ nm uncertainty in the laser frequency.

#### 2.1.2 Index of Refraction Uncertainty

While the index of refraction is extremely close to unity because the depositions are carried out in a vacuum, it is not exactly so because of residual gas in the chamber, and also because of the presence of a very sparse atomic vapor consisting of the atoms being deposited. To obtain a rough estimate of this effect for the NIST depositions, we use the measured background pressure of 1 × 10^−6^ Pa and assume that the residual gas was air. Using a 425 nm air index value of 1.000276 at atmospheric pressure, the correction to the index of refraction is then +2.76 × 10^−15^. To estimate the effect of the Cr vapor, we use the near-resonance expression for the index of refraction[17]

$$n^{2}=1-\frac{2Nf_{0}e^{2}Ä}{V{å}_{0}m{ù}_{0}(4{Ä}^{2}+{Ã}^{2})}$$ (1)

where *N/V* is the number density of Cr atoms, *f*_0_ is the oscillator strength of the transition, *e* is the electron charge, *Δ* is the detuning from resonance, *ε*_0_ is the permittivity of free space, *m* is the electron mass, *ω*_0_ is the resonance frequency and *Γ* is the atomic linewidth. For an estimate of the correction to the index, we can take *N/V* ≈10^15^ m^−3^, *f*_0_ ≈ 1, *Δ* = 2*π* × 500 MHz, and *ω*_0_ = 2*πc/λ* = 4.43 × 10^−15^ s^−1^. For *Γ* we need to take account of power broadening, and so use the expression *Γ* = *Γ*_0_ (1+*I*/*I*_0_)^1/2^, where *Γ*_0_ is the natural linewidth of the atomic transition (2*π* × 5 MHz), *I* is the laser intensity, and *I*_0_ is the saturation intensity for the atomic transition (85 W m^−2^ for the ^7^S_3_ to ^7^P_4_° transition in Cr). Taking a typical value for *I* of 1.2 MW m^−2^, we obtain a correction to the index of refraction of −4.2 × 10^−8^.

Because these corrections are based on rough estimates, we take half the value as a correction, and leave the other half as an uncertainty in the correction. Thus we obtain pitch corrections of (−2.9 × 10^−13^ ± 2.9 × 10^−13^) nm and (+4.5 × 10^−6^ ± 4.5 × 10^−6^) nm for the residual gas and Cr vapor corrections, respectively.

### 2.2 Standing Wave Alignment

**Fig. 2:**
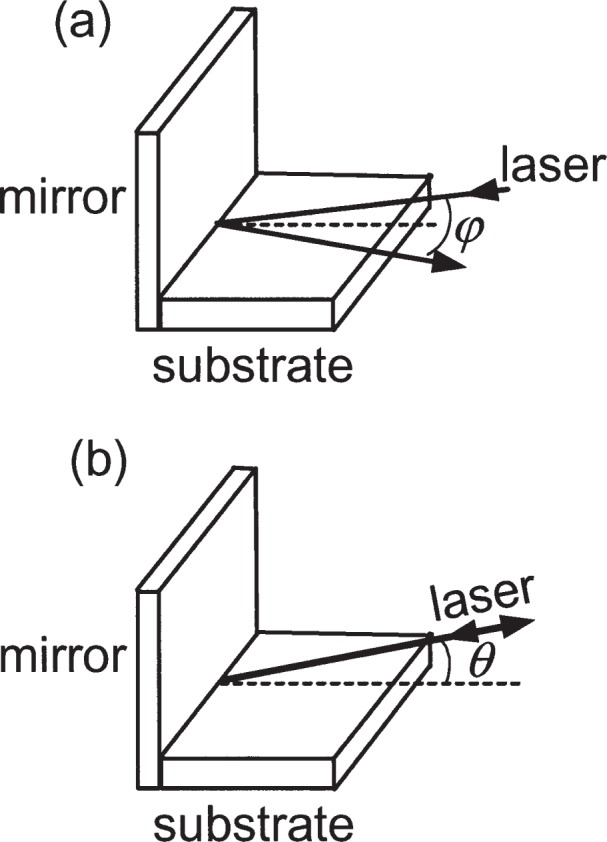
Possible laser misalignments. (a) laser collinearity, (b) substrate alignment.

Going beyond the fundamental uncertainty of the wavelength of the light used for the deposition, the next most obvious source of uncertainty arises because of basic alignment issues. Considering the geometry of the experiment, two errors can arise from misalignment of the laser standing wave. The first of these comes about when the counterpropagating beams that form the standing wave are in a plane parallel to the substrate, but are not exactly collinear (Fig. 2a). If their propagation directions deviate from 180° by an amount *φ*, the resulting pitch on the artifact will be longer than *λ*/2 by a factor 1/cos*φ* ≅ 1+*φ* ^2^/2. In the NIST apparatus, *φ* could be set to 0 with an uncertainty of ± 0.5 mrad, resulting in a pitch correction that could range from 0 to +2.5 × 10^−5^ nm. For the purposes of our error budget we therefore assign to this effect a correction of + 1.3 × 10^−5^ nm with an uncertainty of ± 1.3 × 10^−5^ nm. The second error arises when the laser beams are truly counterpropagating, but do not propagate in a plane parallel to the substrate (Fig. 2b). This will happen, for example, if the mirror that reflects the counterpropagating beam is not perfectly perpendicular to the substrate, or if it is not perfectly flat. If this misalignment is *θ*, the pitch on the substrate will be longer than *λ*/2 by a factor 1/cos*θ* ≅ 1+*θ* ^2^/2. In the NIST apparatus, the mirror was manufactured to a specified flatness of <*λ*_HeNe_/10 (*λ*_HeNe_ is the HeNe laser wavelength 633 nm) over the entire area, so the flatness was not a significant issue. The alignment of the mirror, however, was only accurate to ± 1 mrad. As with the collinearity correction, we use this angular uncertainty to estimate a pitch correction of (+5.3 × 10^−5^ ± 5.3 × 10^−5^) nm.

### 2.3 Wavefront Curvature

**Fig. 3:**
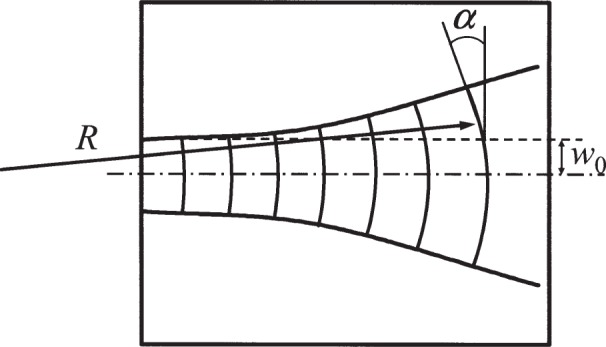
Effect of wavefront curvature on artifact pitch. A Gaussian beam with 1/*e*^2^ waist *w*_0_ travels across the substrate. A wavefront with a radius of curvature *R* makes an angle *α* with respect to the nominal plane wavefront when measured at a distance *w*_0_ from the axis.

In the idealized version of laser-focused atomic deposition, the standing wave is made up of two counterpropagating plane waves. In actuality, the standing wave is formed by reflecting a Gaussian laser beam onto itself using a flat mirror. The waist of the Gaussian beam, where the wavefront is flat, is located as close as possible to the mirror surface. This ensures that the wavefronts of the incoming and outgoing beams will match as well as possible. The result is that the nodes of the standing wave will be spherical, with a radius *R* that varies as a function of distance *z* from the mirror according to *R* = *z*[1+(π*w*_0_^2^/*λz*)^2^], where *w*_0_ is the 1/*e*^2^ radius at the beam waist and *λ* is the laser wavelength [18]. These spherical nodes will result in pitch lines with curvature in the plane of the substrate, and may also cause some asymmetric blurring of the pitch lines as the atoms pass through a curved “channel” on their way to the substrate. An upper limit on the error caused by these effects can be estimated by considering the angle *α* = tan^−1^(*w*_0_/*R*) that the curved pitch lines make, relative to the nominal pitch line direction, at a distance *w*_0_ from the beam axis (see Fig. 3). This angle will result in an effective pitch that is larger by a factor of 1/cos *α* ≅ *α* ^2^/2, if the pitch is measured exactly along the laser propagation direction. For the NIST depositions, *w*_0_ was 0.11 mm, and *z* was at maximum 15 mm. The corresponding radius of curvature is 548 mm, and hence the angle *α* = 0.2 mrad. Thus a pitch measurement taken in some region of the deposited lines would have an error somewhere between 0 and +4.3 × 10^−6^ nm, depending on whether the measurement was done exactly at the center or near one of the corners of the area covered by lines. We take half the maximum value as the correction to the pitch, and use the same value as the uncertainty.

### 2.4 Gaussian Beam Phase Shift (Guoy Phase)

Another effect on the pitch of the deposited lines that stems from the Gaussian nature of the laser beam comes as a result of the fact that a Gaussian beam experiences an axial phase shift when it passes through a focus. This phase shift, sometimes referred to as the Guoy phase, is given by[19]

$$\eta (z)=\tan^{-1}(z/z_{0}),$$ (2)

where *z* is the distance along the axis from the beam waist and *z*_0_ = π*w*_0_^2^/*λ* is the Rayleigh length of the Gaussian beam, *λ* being the wavelength and *w*_0_ the 1/*e*^2^ beam radius at the waist. The result of this phase shift is an effective wavenumber *k′* = *k* − *η* (*z*)/*z* , where *k* = 2π/*λ* is the free-space, plane-wave wavenumber of the laser light. This effective wavenumber leads to an error if a measurement is made by counting pitch lines and assuming the distance is exactly an integral number of free-space plane-wave half wavelengths. If the pitch lines are counted from a point *z*_1_ to a point *z*_2_ (as measured from the beam waist, located at the retroreflecting mirror), the error will be

$$\Delta z=\frac{\lambda }{2\pi }[\tan^{-1}(z_{2}/z_{0})-\tan^{-1}(z_{1}/z_{0})].$$

Inserting the values *λ* = 425.55 nm, *z*_0_ = 89 mm, *z*_1_ = 4 mm, and *z*_2_ = 5 mm from the NIST depositions, we obtain *Δz* = 0.76 nm over a distance of 1 mm, or an effective pitch error of up to +1.6 × 10^−4^ nm, depending on where the measurement is made. As with other corrections, we take half this value as the correction, with the same amount for an uncertainty.

### 2.5 Substrate Temperature

One of the most basic effects that can cause an error in the pitch of the deposited lines is a difference in substrate temperature between when the lines are deposited and when they are used as an artifact. This temperature difference causes a thermal expansion or contraction of the substrate and hence a change in pitch of the lines. In principle it is possible to minimize this effect by performing depositions and subsequent measurements under carefully controlled temperature conditions and using substrate materials with small thermal expansion coefficients. Alternatively, it is possible to correct for thermal expansion by making careful temperature measurements during deposition and subsequent measurement. Correcting, however, always introduces additional uncertainties because of temperature measurement uncertainties and imprecise knowledge of the substrate coefficient of thermal expansion.

For the NIST depositions, no attempt was made to control thermal expansion, but substrate temperature measurements were made during deposition via a thermocouple glued to the sapphire substrate. These measurements showed that the sample temperature was higher than ambient due to radiant heating from the Cr source, and had an average value of (29 ± 0.4) °C, where the uncertainty is one standard deviation of nine temperature measurements taken during the course of the deposition. To this uncertainty, we add a roughly estimated ± 1 °C to account for possible thermal gradients on the sample. Combining these temperature uncertainties in quadrature and using the thermal expansion coefficient for sapphire [(4.8 to 6.3) × 10^−6^ °C^−1^, depending on crystal direction] we calculate an estimated net pitch uncertainty due to thermal effects of ± 0.0013 nm. Additional corrections and uncertainties introduced when making measurements on the artifact at a temperature other than 29 °C are not considered here, but are discussed in Sec. 3.

### 2.6 Substrate Curvature

**Fig. 4:**
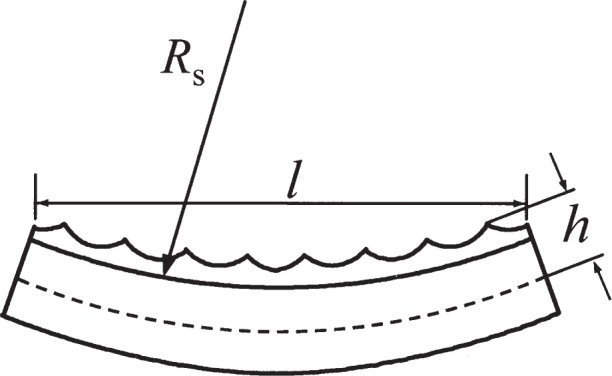
Effect of substrate curvature on artifact pitch. Laser-focused lines are deposited on a substrate with a local radius of curvature *R*_s_ that extends over a region with dimension *l*. The lines are located a distance *h* above the neutral plane of the substrate.

Substrate curvature can arise from a number of sources, including inherent curvature, warping through clamping, and warping due to stress in the deposited Cr film. The effect of this curvature on the pitch of an array of deposited lines can manifest itself in two distinct ways. One effect arises because curvature causes the effective angle between the laser standing wave propagation direction and the local surface tangent to be non-zero, resulting in an error similar to the misalignment error discussed above and shown in Fig. 2b. If the substrate has a local radius of curvature *R*_s_ over a region of lateral dimension *l*, the effective angle *θ* ranges from −*l*/2*R*_s_ to +*l*/2*R*_s_ (see Fig. 4). Thus the pitch can be off by a factor of up to 1/cos(*l*/2*R*_s_) ≅ 1+*l*^2^/8*R*_s_^2^. The other effect on the pitch due to substrate curvature arises because the peaks of the nanostructures are located at some height *h* above the neutral plane of the substrate. This effect only plays a role if the curvature changes between deposition and later utilization, as would happen, for example, if the clamp in the deposition chamber warps the substrate elastically. The correction factor associated with this effect is 1±*h*/*R*_s_, where the plus or minus sign is taken depending on whether the curvature is concave or convex.

#### 2 6.1 Inherent Substrate Curvature

Inherent curvature is of course quite sample-dependent. It can be extremely low, as for example on an optical flat with a specification of *λ*_HeNe_/20 ≈ 30 nm over a 25 mm area, which corresponds to a radius of 2600 m. More typically, the curvature might be on the order of a common specification for silicon wafers, i.e., 10 µm peak-to-valley flatness over a 75 mm diameter region, which corresponds to a radius of 70 m. The curvature can also be more pronounced, as was found to be the case for the sapphire sample discussed in detail in Sec. 3. Here, the radius of curvature was measured to be (0.91 ± 0.05) m. Using these three different curvatures, we can estimate the error due to effective angle, using *l* = 1 mm, to be +3.9 × 10^−12^ nm, +5.4 × 10^−9^ nm, and +3.2 × 10^−5^ nm, respectively. Since we are estimating corrections and uncertainties for the NIST depositions, we take half the 0.91 m curvature value as a correction and use the same amount as an uncertainty. The error due to nanostructure height does not play a role here, as the curvature is assumed to be permanent.

#### 2.6.2 Substrate Warping

Warping of the substrate during clamping is also very situation-dependent. A very rough upper estimate of a pitch error that might arise can be made if we assume the sample is clamped at both ends over a 1 µm irregularity in the mount. Assuming a 10 mm overall sample size, the result is a radius of curvature of 12.5 m. Using *l* = 1 mm and *h* = 0.25 mm, the corresponding pitch error due to effective angle is (+8.5 × 10^−8^ ± 8.5 × 10^−8^) nm and the uncertainty due to nanostructure height is ± 0.0043 nm.

#### 2.6.3 Film Stress Induced Curvature

To estimate the effects of film stress-induced curvature, we can use the Stoney formula for the radius of curvature of a substrate-film system in terms of the mechanical properties of the substrate and film materials [20]:

$$R=\frac{1}{6\sigma_{f}}\frac{E_{s}d_{s}^{2}}{(1-\nu_{s})d_{f}},$$ (4)

where *R* is the stress-induced radius of curvature, *σ*_f_ is the film stress, *E*_s_ is Young’s modulus for the substrate, *d*_s_ is the thickness of the substrate, *ν*_s_ is Poisson’s ratio for the substrate, and *d*_f_ is the film thickness. For an example of the magnitude of this effect, we can apply this formula to the specific sample discussed in Sec. 3, which had a substrate thickness of 0.5 mm and an average film thickness of about 100 nm. Using the bulk properties *E*_s_ = 380 GPa and *ν*_s_ = 0.24 for the sapphire substrate [21], and a value of 1 GPa for the film stress in a thermally deposited Cr film [22], we calculate a radius of curvature of 208 m. This radius yields a pitch error due to effective angle of (+3.1 × 10^−10^ ± 3.1 × 10^−10^) nm using *l* = 1 mm. Assuming the substrate is clamped during deposition and then warps only when released, the error arising from the height *h* above the neutral plane becomes (−6.0 × 10^−7^ ± 6.0 × 10^−7^) nm.

### 2.7 Atom Beam Divergence

The final effect on the deposited line pitch that we consider stems from the fact that the atom beam entering the standing wave has in general some degree of divergence. We can estimate the effect of this divergence if we consider the standing-wave-induced focusing of atoms in a purely paraxial model. It should be noted, though, that usually the focusing of the atoms has a fairly significant contribution from “channelling,” or multiple crossovers of the atom trajectories as they approach the substrate through the standing wave [24]. This channeling significantly reduces the effect of atom beam divergence, so the estimate given here is most likely an overestimate.

**Fig. 5:**
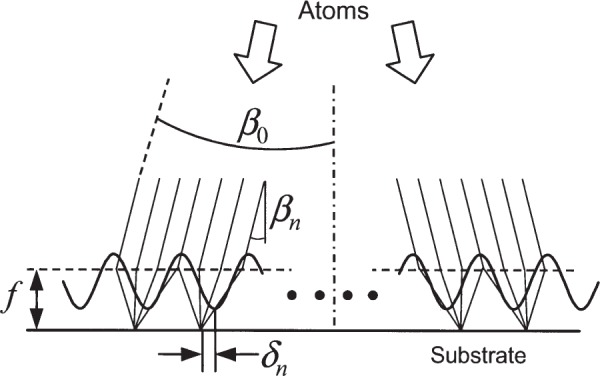
Effect of atomic beam divergence on artifact pitch. Atoms in an atomic beam with overall divergence half angle *β*_0_ are focused by the light force lenses formed by the nodes of a laser standing wave with atom-optical focal length *f*. At the *n*th node of the standing wave, the focal spot deviates from the expected focal position by an amount *δ_n_* = *fβ_n_*.

In an atom beam with divergence half-angle *β*_0_, atoms enter the standing wave at angles ranging from +*β*_0_ relative to the surface normal at one extreme of the artifact to −*β*_0_ at the other. This varying angle of incidence can be written as *β_n_* = 2*β*_0_*n*/*N*, where *N* is the total number of lines in the artifact and *n*, ranging from −*N*/2 to +*N*/2 , corresponds to the *n*th node of the standing wave. For paraxial optics, if a bundle of rays are parallel but incident at an angle *β_n_* relative to the axis, the focal spot is shifted off the axis by an amount *δ*_n_ = *fβ_n_* , where *f* is the focal length of the light-force lens that focuses the atoms [23] (see Fig. 5). Thus the pitch error encountered in measuring between adjacent lines will be Δ*p* = *f* (*β*_n+1_ − *β_n_*) = 2*fβ*_0_/*N*. Writing *N* = *w*/*p*, where *w* is the width of the artifact and *p* is the pitch, we obtain Δ*p* = 2*fβ*_0_*p*/*w*. For the NIST depositions, we can take *f* to be approximately equal to the laser beam waist, or 0.11 mm, *β*_0_ ≈ 0.08 mrad, and w ≈ 1 mm, resulting in a maximum pitch error of +0.0037 nm at the edges of the deposition. Thus we take the pitch correction to be (+0.0019 ± 0.0019) nm.

## 3. Diffraction Measurements of a Prototype Pitch Artifact

Based on the considerations of the previous section, it appears that the average pitch of a laser-focused atomic deposition artifact should be given by half the vacuum wavelength of light tuned to an atomic resonance with an accuracy of ± 0.0049 nm or better. In order to provide an experimental test of this assertion, we have measured the average pitch of a Cr sample by observing the diffraction of 351.1 nm laser light from an argon ion laser. These measurements show good agreement between the expected and actual pitch within the stated uncertainty, adding confidence to our belief that laser-focused atomic deposition can produce robust nanoscale pitch standards.

### 3.1 Artifact Description

**Fig. 6:**
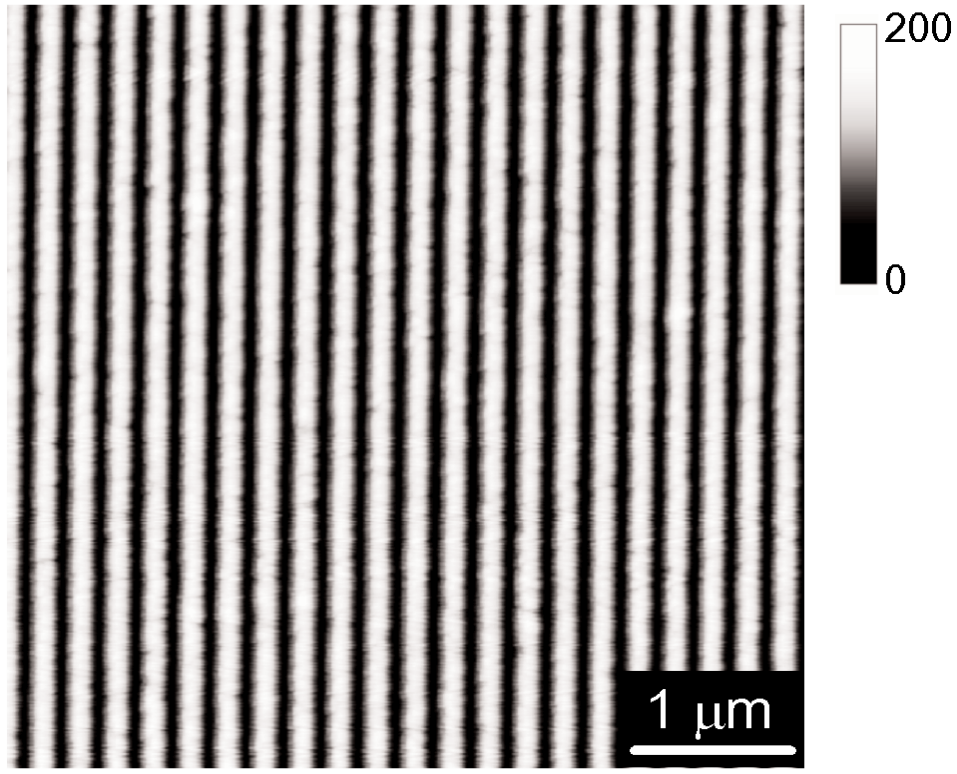
Atomic force microscope image of a 5 m × 5 m region of the artifact used in the diffraction measurements.

The artifact used in our diffraction measurements was produced by laser-focused atomic deposition of Cr according to methods described generally in [3] and more specifically in [24]. For the artifact discussed here, the Cr atomic beam was mechanically collimated with a 0.3 mm × 1 mm slit and transversely cooled to a half-angle divergence of 0.08 mrad. Laser focusing was carried out in a standing wave of detuning 500 MHz propagating parallel to the 1 mm dimension of the atom beam with single beam power 66 mW and 1/*e*^2^ radius 0.11 mm. A number of adjacent depositions were carried out on a polished sapphire substrate with dimensions 4 mm × 10 mm × 0.5 mm, creating a 1 mm × 3.6 mm column of gratings with lines running along the long direction. Deposition times varied from 35 min to 182 min, yielding Cr pads with peak-to-valley heights ranging from 30 nm to 140 nm and line widths ranging from 55 nm to 120 nm. Because it gave the clearest diffraction peak, the deposition with the largest peak-to-valley height was used for the studies described here. An AFM image taken on this sample is shown in Fig. 6.

### 3.2 Experimental Setup

**Fig. 7:**
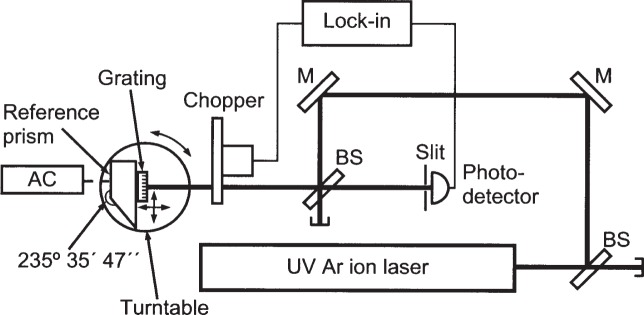
Schematic of diffraction experiment, showing ultraviolet Ar ion laser, beamsplitters (BS), mirrors (M), grating, reference prism, autocollimator (AC), turntable, slit, photodetector, lock-in amplifier, and chopper.

A schematic diagram of the diffraction measurement setup is shown in Fig. 7. In essence, a Littrow configuration [25] was used to measure the first order diffraction angle of an ultra-violet (UV) Ar ion laser line. Using an autocollimator and a reference prism, this angle was measured with a statistical standard uncertainty of better than 1″ and a combined standard uncertainty of ± 9″.

Laser light was supplied by an Ar ion laser producing several lines in the mid-UV region (333.6 nm to 363.8 nm) with a total power of approximately 2.5 W. Of the lines in the laser beam, only the strongest at 351.1 nm was used for the diffraction measurements. The laser beam was sampled by a beamsplitter reflecting nominally 9 % of the laser power, and the reflected beam was directed towards the grating through a second 9 % beamsplitter. At the grating, the laser beam was measured to have a very nearly round, Gaussian profile with 1/*e*^2^ radius of (2.0 ± 0.1) mm. The grating was located at a total optical distance of 4.3 m from the approximate beam waist location within the laser, and hence was within the Rayleigh length of the Gaussian laser beam. The wavefront radius of curvature at the grating was calculated from the beam diameter and waist location to be 12 m.

The grating was mounted on an *x–y* stage, which was in turn mounted on a precision turntable. Also mounted on the turntable was a reference prism with two mirrored faces ground at an external angle of 235°35′47″ [26]. This angle was chosen because it causes the surface normals of the two mirrored faces to make an angle very close to the expected angle for first order Littrow diffraction of 351.119 nm light from a grating with the expected pitch.

Light reflected from the grating returned along the path of the incoming laser beam, passed through the beamsplitter, and was incident upon a photodetector fitted with a 0.1 mm slit, located a distance of 550 mm from the grating. The photodetector signal was detected with a lock-in amplifier, which was referenced to a chopper operating at 227 Hz placed in the laser beam just in front of the grating.

Diffraction measurements were conducted under ambient laboratory conditions, i.e., at an average temperature of 22 °C. Since this differs from the deposition temperature of 29 °C, the expected pitch must be corrected from the nominal value of 212.7787 nm. Using a mean value for the sapphire thermal expansion coefficient of 5.5 × 10^−6^ °C^−1^, we obtain an expected pitch of 212.7705 nm.

In order to make accurate measurements of the diffraction angle, a number of adjustments were made to ensure the alignment of the apparatus. First, the incident laser beam was adjusted to travel parallel to the laser table, and the turntable axis of rotation was adjusted to be perpendicular to the laser table (both within 1 mrad). Then the laser was aimed to pass symmetrically through the axis of rotation of the turntable. To accomplish this we first mounted a 75 µm diameter wire vertically on the turntable and moved it onto the axis of rotation by adjusting its position with the *x–y* stage while observing with a measuring telescope and rotating through 360°. Then the laser beam was directed at the wire while observing scattered light with a photodetector. By maximizing the scattered light, the laser could be centered on the axis of rotation with an accuracy of approximately ± 50 µm. With the measuring telescope still aimed at the axis of rotation, the grating was then observed while rotating the turntable through 90° and 180°. Using the *x–y* stage, the grating was translated until its face was visually centered and its surface was as close as possible to the axis of rotation. Based on the visual resolution of the measuring telescope, this was accomplished with an accuracy of approximately ± 50 µm. As a final alignment, the turntable was rotated so that the specular reflection from the grating returned along the incident beam, and the photodetector with the 0.1 mm slit was positioned so that the signal was maximized. This established the Littrow condition with an accuracy of approximately ± 1 mrad.

**Fig. 8:**
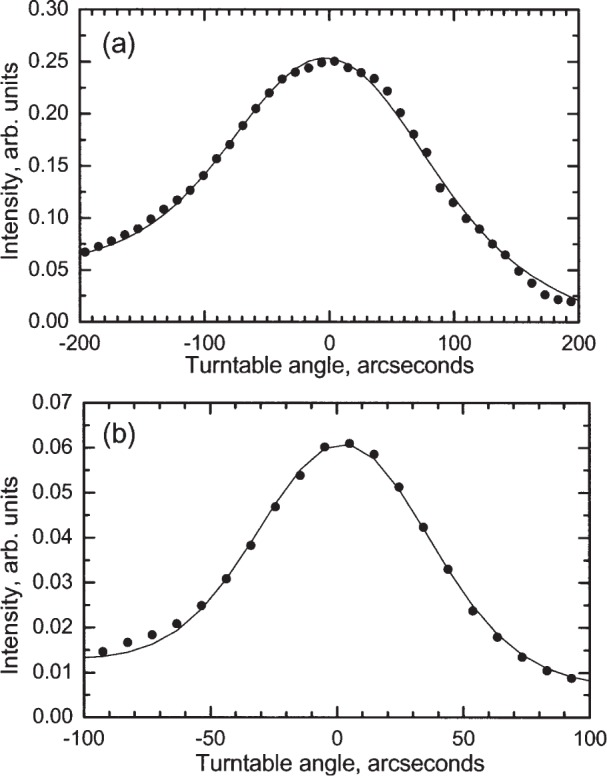
Examples of measured intensity vs turntable angle. (a) specular peak; (b) diffracted peak. Solid circles show measurements, while solid lines show the fit to a Gaussian with a quadratic background used to extract the peak center.

Measurements were conducted by rotating the turntable through an angular scan of approximately 600″ in increments of 10″ while the photodetector signal was recorded. This rotation was accomplished with a closed-loop motion control system that had a repeatability of approximately one arcsecond. Examples of such measurements are shown in Fig. 8.

A measurement of the diffraction angle consisted of (1) conducting an angular scan of the specular beam; (2) fitting a Gaussian function with quadratic background to the data and extracting a center, which could be done with a statistical accuracy of ± 0.7″; (3) rotating the turntable to the center as determined by the fit; (4) zeroing the autocollimator on one of the reference prism faces; (5) rotating the turntable to the first order diffraction position and conducting an angular scan; (6) fitting a Gaussian function and extracting the center, as with the specular beam, though with a slightly larger statistical uncertainty of ± 1″; (7) rotating the turntable to the center as determined by the fit; and (8) observing the other face of the reference prism with the autocollimator, and hence determining the deviation, if any, from the prism angle of 55°35′47″.

As an additional check against unknown systematic errors, measurements were made with the grating rotated both clockwise and counterclockwise when viewed from above. In order to ensure that measurements were done on the central portion of the grating, a series of measurements was conducted while translating the grating parallel to its surface in a direction perpendicular to the lines in increments of 0.16 mm. The central section of the grating was assumed to be the area with the brightest diffraction signal, and the four measurements closest to this region were recorded. The results of these measurements are shown in Table 2, where their average and standard deviation are also shown. Using the average measured diffraction angle of 55°35′49.3″, we can use the Littrow formula sin*θ* = *λ*/(2*p*), where *θ* is the diffraction angle, *λ* is the wavelength of the UV light, and *p* is the pitch, to calculate a measured pitch of 212.7777 nm.

### 3.3 Pitch Measurement Uncertainties

In this section we discuss the uncertainties associated with the measurement of the pitch of the Cr artifact by diffraction as discussed in the preceding section. As with the correction and uncertainty estimates given for the artifact fabrication process, an attempt has been made to be thorough, but as always it is possible that a source of error has been overlooked. The uncertainties discussed here are summarized in Table 3.

#### 3.3.1 Temperature Effects

The diffraction measurements were carried out in a laboratory that was not especially controlled for temperature variations. As a result, the temperature at which the diffraction measurements were conducted is known only to have been in the range 20 °C to 24 °C, the typical variation for the laboratory space used. Because the temperature did not change significantly over the course of a measurement, and because temperature dependence of the calibration of the instruments used did not play a significant role, temperature uncertainty did not affect the measured diffraction angles in any significant way. However, because the ambient temperature was different from the deposition temperature, two uncertainties are introduced into the actual pitch of the grating during measurement. The first of these arises from the ± 2 °C uncertainty in temperature, which together with the mean sapphire thermal expansion coefficient of 5.5 µm^−1^ °C^−1^ results in an uncertainty of ± 0.0023 nm in the final pitch measurement. The second comes from the fact that the sapphire thermal expansion coefficient is not known exactly, and in fact varies from 4.8 × 10^−6^ °C^−1^ to 6.3 × 10^−6^ °C^−1^, depending on crystal structure and orientation [21]. This leads to an uncertainty in the correction factor used to predict the actual pitch, and hence to an uncertainty in the final pitch measurement of ± 0.0011 nm.

### 3.3. Laser Wavelength Uncertainty

The 351.1 nm laser line has been measured to have a vacuum wavenumber of 28 472.568 cm^−1^ with a stated absolute uncertainty of ± 0.002 cm^−1^ [27]. Propagating this uncertainty through the Littrow equation results in a contribution of ± 1.5 × 10^−5^ nm to the pitch measurement uncertainty. Since the measurements were performed in ambient laboratory air, there is also an uncertainty associated with the index of refraction. To estimate this, we take the range of environmental extremes expected for the laboratory and calculate the effect on the index of refraction via the Edlén equation [28]. Assuming a temperature range of 20 °C to 24 °C, a relative humidity range of 20 % to 80 %, and a barometric pressure range of 98.8 kPa to 100.2 kPa, we obtain an average air wavelength for our conditions of 351.1192 nm with an uncertainty of ± 0.0014 nm. This wavelength uncertainty translates into a pitch uncertainty of ± 8.5 × 10^−4^ nm.

#### 3.3.3 Angle Measurement Uncertainties

In this section we discuss angular uncertainties associated with the measurement of the first-order diffraction angle. Using propagation of error through the Littrow equation, these uncertainties result in a pitch uncertainty of δ*p* = |*p* cot*θ*_0_ δ*θ* |, where δ*θ* is the angle uncertainty (in radians) and *θ*_0_ is the Littrow angle. For the present case, where *θ*_0_ = 0.9703 rad, δ*p*/*p* = 0.685|δ*θ* |.

##### 3.3.3.1 Reference Prism Accuracy

As previously mentioned, the reference prism external angle was measured with an expanded (*k* = 2) uncertainty of ± 0.6″ [26]. This contributes a one-standard deviation uncertainty of ± 2.1 × 10^−4^ nm to the pitch uncertainty. The mirrored prism faces were manufactured to be perpendicular to the base with a stated specification of ± 1′. The mounting of the prism with respect to the axis of rotation of the turntable was such that these were parallel to within ± 1 mrad. If either of these vertical alignments has a magnitude of *γ*, the measured angle will have a relative error ± (1−cos *γ*) ≅ ± *γ* ^2^/2. The result is an angular uncertainty of ± 0.008″ from the prism specification and ± 0.1″ from the alignment. These become pitch uncertainties of ± 6.0 × 10^−6^ nm and ± 7.1 × 10^−5^ nm, respectively.

##### 3.3.3.2 Grating Alignment

By observing the reflected and diffracted laser spots as the turntable was rotated, the grating was aligned so that both its face and the grating lines were parallel to the axis of rotation with an accuracy of ± 1 mrad. Given a Littrow configuration, the actual measured diffraction angle *θ*_meas_ can be expressed in terms of any misalignments of this type as

$$\sin\theta_{\text{meas}}=\frac{\lambda \cos\varphi }{2p\cos\alpha },$$ (5)

where *φ* is the rotation of the grating lines (i.e., the azimuth of the grating) and *α* is the tilt relative to the axis of rotation. From this it can be seen that *θ*_meas_ will have an error between 0 and −0.15″ from *φ* and between 0 and +0.15″ from *α*. Combining these, we obtain a pitch uncertainty of ± 1.1 × 10^−4^ nm.

Another alignment uncertainty arises from the degree to which the exact Littrow condition is achieved, as determined by whether the normal and diffracted beams propagate exactly back along the incident beam direction. If the photodetector slit position is such that the normal and diffracted beams make a small angle *β* with respect to the incoming beam, it can be shown that the measurement of the diffraction angle will have an error (*β*^2^/8)tan*θ*_0_, where *θ*_0_ is the Littrow angle. Given the alignment uncertainty of ± 1 mrad, this error will cause the diffraction angle to have an uncertainty of ± 0.04″, which corresponds to a pitch uncertainty of ± 2.1 × 10^−5^ nm.

##### 3.3.3.3 Autocollimator Calibration and Repeatability

The autocollimator used was a visual telescope model with a total range of 10′ and least count gradation of 0.2″. Repeated measurements showed that using the visual scale, an angle could be determined within ± 0.15″ (one standard deviation random uncertainty). All measurements were conducted within ± 10″ of the center of view, so any contributions to the uncertainty from scale calibration were insignificant. Thus the autocollimator uncertainty contributed ± 1.1 × 10^−4^ nm to the pitch uncertainty.

##### 3.3.3.4 Angle Setting

Two additional sources of uncertainty arose from the measurement protocol, which involved measuring intensity as a function of angle, finding the center with a least-squares fit to a Gaussian, and then moving the turntable to this center. For each peak, then, there was a statistical error from the center finding and a repeatability error from the turntable motion. For the specular peak, the center finding typically had a one-standard deviation of ± 0.7″, while for the diffracted peak it was a little larger at ± 1″ because the intensity was less. For both peaks the motion repeatability was ± 1″. These angular uncertainties translate into pitch uncertainties of ± 4.9 × 10^−4^ nm and ± 7.1 × 10^−4^ nm, respectively.

##### 3.3.3.5 Grating Curvature and Alignment

As it turns out, the most significant source of uncertainty in the diffraction angle measurements described here was a result of a combination of grating curvature and an uncertainty in locating the incident laser beam and grating surface on the turntable axis of rotation. If the grating were perfectly flat and infinite in extent, any displacement relative to the axis of rotation would not introduce any error, as this would not change the rotation angle of the grating surface. However, a slightly curved grating that is not on axis and/or is illuminated by a finite laser beam not aimed at the axis will cause a redirection of the reflected or diffracted beam as the turntable is rotated. To visualize this effect, it is useful to recognize that this situation is analogous to a lens that is being moved transversely to its axis of symmetry, the result of which is a change in angle of the transmitted beam.

To estimate the magnitude of this effect, we first estimated how much the grating was displaced relative to the incident laser beam when the turntable was rotated through the Littrow angle. It can be shown that if the laser misses the rotation axis by an amount *L_x_* and the surface of the grating is a distance *L_y_* away from the rotation axis, the grating will translate an amount

$$\left|\Delta |=|2\sec\theta \sin(\theta /2)[L_{x}\sin(\theta /2)+L_{y}\cos(\theta /2)]\right|$$ (6)

when the turntable is rotated through an angle *θ*. Evaluating this expression with *L_x_* = *L_y_* = 0.05 mm and *θ* = ± 0.9703 radians results in a maximal value of | *Δ* | of 0.11 mm. Next, we made a measurement of the radius of curvature of the grating by positioning the turntable to observe the specular beam while translating the grating. This showed an approximate radius of curvature of (0.91 ± 0.05) m. Finally, we performed a numerical calculation of the propagation of a laser beam through a lens of focal length 455 mm (equivalent to a mirror with radius 910 mm) as a function of displacement from the axis. The laser beam used in the calculation was Gaussian with beam parameters as measured in the experiment, with the additional condition that it was clipped to a width of 0.56 mm, as it would be for a 1 mm grating viewed at an angle of 55°35′47″. For small displacements we found that this calculation showed a very linear angular shift as a function of lens displacement, with a coefficient of −59″ mm^−1^, given by a least squares fit. Using this coefficient with the estimated value for *Δ*, we arrive at a net angular uncertainty of ± 6.5″ with a corresponding pitch uncertainty of ± 0.0046 nm.

#### 3.3.4 Measurement Standard Deviation

As discussed in Sec. 3.2, a number of measurements of the diffraction angle were obtained under what should have been nominally equivalent conditions, that is, at slightly different grating positions and with the turntable rotated either clockwise or counterclockwise. These measurements did not give identical results, and so can be used to provide an estimate of additional, unaccounted for errors. We calculate the standard deviation of these measurements, and consider this to be a type A uncertainty to be added in quadrature to the other uncertainties.

## 4. Conclusion

In this paper we have studied a range of possible error sources that could contribute to the uncertainty in the average pitch of an artifact fabricated by laser-focused atomic deposition. Based on this analysis, we believe a Cr sample fabricated on sapphire at NIST in February 1998 has an average pitch of (212.7787 ± 0.0049) nm, provided the substrate temperature is 29 °C. We have also conducted diffraction measurements in order to confirm our belief. These show an average pitch of (212.7777 ± 0.0069) nm, in good agreement with the expected pitch of (212.7705 ± 0.0049) nm at 22 °C.

The main conclusion to be drawn from this work is that laser-focused atomic deposition has potential as a means of fabricating nanometer-scale pitch standards whose average pitch is traceable to an absolute atomic frequency with a relative uncertainty of a few times 10^−5^. Because they can be fabricated from stable, hard materials such as Cr, these pitch standards could find use in calibrating various nanometer-scale measuring instruments.

One potential drawback that laser-focused atomic depositions samples appear to have is a certain amount of roughness in the deposited lines (see Fig. 6). Thus while the average pitch is extremely accurate, a measurement conducted on a single pair of lines will generally have a much greater uncertainty. Depending on how the pitch standard is employed, this may limit the usefulness in some cases. However, in many other cases it is possible to average in such a way as to reduce the random error associated with line roughness. Furthermore, refinements in the fabrication process that reduce line roughness may become available with further research. For example, a certain amount of smoothing has been observed during reactive ion etching of Cr lines on Si [11].

As research progresses in this field, we can expect refinements and extensions to develop that could widen the usefulness of these artifacts. For example, it has already been shown that lines with a pitch of *λ*/8, or 53.2 nm, can be made by using polarization gradients in the standing wave [8]. Also, a beating, or Moiré, pattern with pitch 44.46 µm can be made by superimposing depositions at two wavelengths [12]. Examining these and other extensions from a metrological point of view promises to yield a wide range of nanoscale calibration artifacts that are traceable to atomic frequencies.

## Figures and Tables

**Table 1:** Corrections and uncertainties associated with deposition of Cr pitch artifact on sapphire in NIST apparatus. Uncertainties are of type B and are intended to be interpreted as 1 standard deviation. The total correction is the sum of all corrections, while the total uncertainty is the quadrature sum of all uncertainties. The expected pitch is *λ*/2 = 212.7766 nm plus the total correction

Source	Pitch correction (nm)	Pitch uncertainty (nm)
Wavelength effects		
1. Acousto-optic modulator RF accuracy	–	± 2.3 × 10^−8^
2. Laser lock accuracy	–	± 6.0 × 10^−7^
3. Absolute atomic resonance accuracy	–	± 4.5 × 10^−5^
4. Index correction for residual gas	−2.9 × 10^−13^	± 2.9 × 10^−13^
5. Index correction for Cr vapor	+4.5 × 10^−6^	± 4.5 × 10^−6^
Standing wave alignment		
1. Colinearity (*φ*)	+1.3 × 10^−5^	± 1.3 × 10^−5^
2 Alignment with substrate (*θ*)	+5.3 × 10^−5^	± 5.3 × 10^−5^
Wavefront curvature	+2.1 × 10^−6^	± 2.1 × 10^−6^
Guoy phase	+8.1 × 10^−5^	± 8.1 × 10^−5^
Substrate temperature	Measurement dependent	± 0.0013
Substrate curvature		
1. Inherent - effective angle	+1.6 × 10^−5^	± 1.6 × 10^−5^
3. Clamp warping - effective angle	+8.5 × 10^−8^	± 8.5 × 10^−8^
4. Clamp warping - nanostructure height	–	± 0.0043
5. Film stress - effective angle	3.1 × 10^−10^	± 3.1 × 10^−10^
6. Film stress - nanostructure height	−6.00 × 10^−7^	6.00 × 10^−7^
Atom beam divergence	+0.0019	± 0.0019
Total	+0.0021	± 0.0049
Expected pitch at 29 °C: (212.7787 ± 0.0049) nm		

**Table 2:** Summary of diffraction angle measurements as a function of lateral grating position and turntable rotation clockwise (CW) or counterclockwise (CCW). Deviation is the measured deviation from the reference prism angle of 55°35′46.9″

Grating position (mm)	Turntable	Deviation″	Diffraction angle
0.16	CW	−5.3	55°35′41.6″
	CCW	+9.2	55°35′56.1″
0.32	CW	+3.2	55°35′50.1″
	CCW	+5.3	55°35′52.2″
0.48	CW	+3.6	55°35′50.5″
	CCW	−3.0	55°35′43.9″
0.64	CW	+10.4	55°35′57.3″
	CCW	−4.2	55°35′42.7″

		Average	55°35′49.3″
	Standard deviation		6.0″

**Table 3:** Uncertainties associated with pitch measurement by diffraction of a UV Ar ion laser line. The measured value was (212.7777 ± 0.0069) nm. All uncertainties are type B standard uncertainties, with the exception of the center fits and measurement standard deviation, which are type A

Source	Angle uncertainty (″)	Pitch uncertainty (nm)
Temperature effects		
1. Ambient temperature		± 0.0023
2. Correction factor		± 0.0011
UV laser wavelength		
1. Absolute accuracy		± 1.5 × 10^−5^
2. Index of refraction		± 8.5 × 10^−4^
Angle uncertainties		
1. Reference prism accuracy	± 0.3	± 2.1 × 10^−4^
1. Reference prism vertical squareness	± 0.008	± 6.0 × 10^−6^
2. Reference prism vertical alignment	± 0.1	± 7.1 × 10^−5^
3. Grating azimuth and tilt	± 0.15	± 1.1 × 10^−4^
4. Deviation from Littrow	± 0.04	± 2.1 × 10^−5^
5. Autocollimator repeatability	± 0.15	± 1.1 × 10^−4^
6. Center fit, specular beam	± 0.7	± 4.9 × 10^−4^
7. Center fit, diffracted beam	± 1	± 7.1 × 10^−4^
8. Turntable repeatability, specular beam	± 1	± 7.1 × 10^−4^
9. Turntable repeatability, diffracted beam	± 1	± 7.1 × 10^−4^
10. Grating curvature with misalignment	± 6.5	± 0.0046
Measurement standard deviation	± 6.0	± 0.0042
Total (quadrature)	± 9.0	± 0.0069
